# The anti-apoptotic and antioxidant effects of Naringenin in varicocele-induced testicular damage

**DOI:** 10.5935/1518-0557.20250169

**Published:** 2026

**Authors:** Zeinab Sadat Mirshaby, Nesa Karimi Nasersarai, Zeinab Soleimany, Setareh Dehkhodaei, Maryam Abbasi, Soroush Taherkhani, Hossein Eyni

**Affiliations:** 1 Department of Biology, Shahre-Qods Branch, Islamic Azad University, Tehran, Iran; 2 Department of Pathobiology, Islamic Azad University, Tehran, Iran; 3 Department of Biology, Tehran North Branch, Islamic Azad University, Tehran, Iran; 4 Department of Cell and Molecular Biology, Faculty of Biological Sciences, Kharazmi University, Tehran, Iran; 5 Zhino-Gene Research Services Co, Tehran, Iran; 6 Department of Physiology, School of Medicine, Iran University of Medical Sciences, Tehran, Iran; 7 Student Research Committee, Iran University of Medical Sciences, Tehran, Iran; 8 Department of Anatomy, School of Medicine, Iran University of Medical Sciences, Tehran, Iran; 9 Stem Cell and Regenerative Medicine Research Center, Iran University of Medical Sciences, Tehran, Iran

**Keywords:** varicocele, Naringenin, apoptosis, oxidative stress, inflammation

## Abstract

**Objective:**

Varicocele is a prevalent condition among the male population, representing a significant cause of male infertility. Naringenin, a flavonoid, has demonstrated significant antioxidant and anti-apoptotic properties.

**Methods:**

In this study, 24 male Wistar rats were divided into four groups: control group, sham group, a varicocele-induced rat (VCL) group, and a varicocele-with-Naringenin (20 mg/kg) treatment group (N20+C). Following a 21-day period, the rats were euthanized, and the quality of the tissue, the level of oxidative stress, the expression levels of HSP70, and the expression levels of the genes VEGF, BCL-2, caspase-3, and IL-6 in the testes were evaluated.

**Results:**

Based on H&E images, varicocele induced tissue damage was improved by Naringenin. The expression of HSP70 in the VCl group increased in comparison to the Sham group, and in the N20+C group (*p*<0.001) it was lower than in the VCL group (*p*<0.05). MDA in the VCL group increased, SOD and TAC decreased when compared to the Control group (*p*<0.01), and there was no significant difference between the N20+C and the Control group. In the VCL group the expression of VEGF (*p*<0.05), caspase-3 (*p*<0.001) and IL-6 (*p*<0.001) genes increased in the VCL Group, and BCL-2 (*p*<0.05) decreased in comparison to the Control group. The expression of VEGF (*p*<0.05) and BCL-2 (*p*<0.05) increased and caspase-3 (*p*<0.001) and IL-6 (*p*<0.001) genes decreased in the N20+C group when compared to the VCL group.

**Conclusions:**

Naringenin has been demonstrated to reduce oxidative stress and apoptosis through the intrinsic pathway in rats with varicocele. This finding suggests that naringenin may be a promising candidate for mitigating the adverse effects associated with varicocele.

## INTRODUCTION

Varicocele is a common condition characterized by the dilation of the veins in the pampiniform plexus, which drains the testes. Totally, 15% of all men suffer from varicocele (Agarwal *et al*., 2007), the left testes involvement is more prevalent and have bigger varicoceles; and 50% of them have bilateral varicocele (Abdulmaaboud *et al*., 1998). The prevalence of varicocele has been documented to be approximately 35% among men experiencing primary infertility, and as high as 81% among those with secondary infertility. Additionally, varicocele has been identified as a risk factor for hypogonadism (Gorelick & Goldstein, 1993). The condition has been shown to cause atrophy and discomfort in the testes, as well as negatively impact male fertility and semen parameters (Ficarra *et al*., 2012). While the etiology of varicocele is understood to be multifactorial, variations in the drainage patterns of the left and right testicular veins have been identified as significant contributing factors to its development (Abdulmaaboud *et al*., 1998); the valves of the testicular venous system can become dysfunctional or damaged, resulting in retrograde blood flow. This condition can lead to increased pressure on the renal vein, which is located between the aorta and the superior mesenteric artery. Consequently, this can elevate hydrostatic pressure within the testicular venous system. The result of these processes is the dilation of the venous plexus in the spermatic cord, ultimately leading to the formation of a varicocele (Naughton *et al*., 2001). Furthermore, physical activity has been shown to contribute to the development of varicocele. Research indicates that engaging in physical activity during puberty may lead to the onset of varicocele in men. Additionally, in older age men, the severity of varicocele tends to increase with higher levels of physical activity (Rigano *et al*., 2004). The exact mechanism by which varicocele contributes to infertility has yet to be fully understood. However, several potential mechanisms have been proposed, including hypoperfusion leading to hypoxia, heat stress, oxidative stress, hormonal imbalances, and exposure to exogenous toxicants (Eisenberg & Lipshultz, 2011).

Naringenin is one of the predominant natural flavonoids, known for its numerous biological and pharmacological effects. The primary sources of naringenin include various edible fruits, such as citrus species, grapes, and tomatoes (Venkateswara Rao *et al*., 2017). The chemical name of Naringenin is 2,3-dihydro-5,7-dihydroxy-2-(4-hydroxyphenyl)-4H-1-benzopyran-4-one and its molecular weight is 272.26 (C_15_H_12_O_5_) (Salehi *et al*., 2019). Naringenin has a wide range of biological activities in the body, including the regulation of inflammation and the treatment of various inflammation-related diseases, such as sepsis, endotoxic shock, fibrosis, and cancer (Zeng *et al*., 2018). Naringenin inhibits apoptosis and reduce oxidative stress (Wang *et al*., 2017); it can reduce free radicals, such as reactive oxygen species (ROS), and increase antioxidant agents, including glutathione (GSH), catalase, and superoxide dismutase (SOD) in chronic diseases such as cardiovascular disease, neurodegenerative disorders, diabetes, lung disease, cancer, and nephropathy (Zaidun *et al*., 2018). Naringenin can reduce the oxidative stress induced by exogenous agents such as H_2_O_2_ in the testes (Sahin *et al*., 2017), and in some animal model that destroys the testes, the administration of naringenin reduced testicular malondialdehyde (MDA) levels, the tumor necrosis factor-α/interleukin-10 ratio, and caspase-3 activity. It also improved overall antioxidant status and decreased histopathological injury in the testes (Fouad *et al*., 2019).

**Figure 1:**
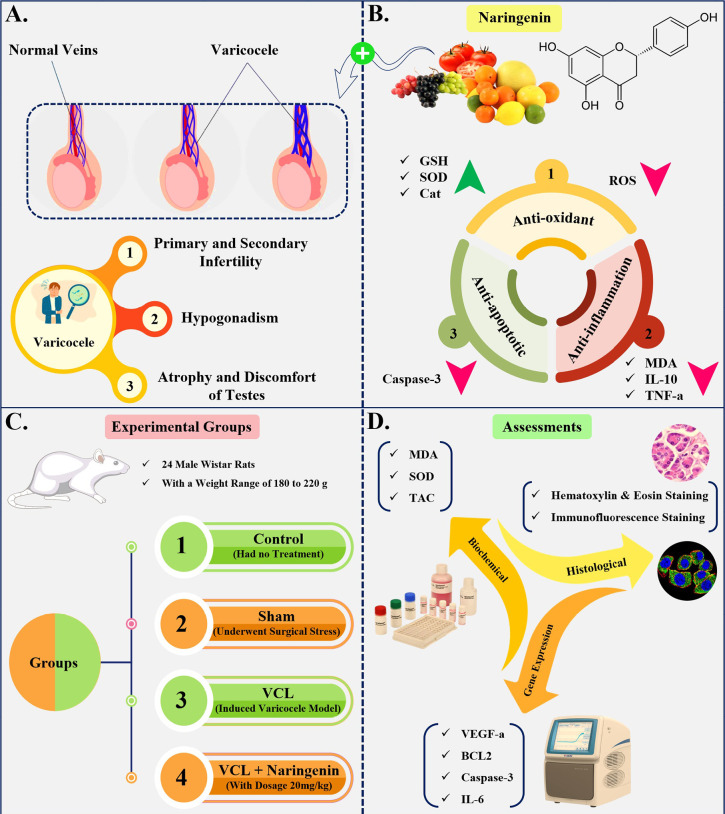
Schematic study diagram.

In the present study, we investigated the protective effects of Naringenin against apoptosis and oxidative stress induced by varicocele in rats. This research is motivated by the high prevalence of varicocele in men, the complications associated with this condition, and its impact on fertility and quality of life, as well as the therapeutic potential of Naringenin (Figure 1).

## METHOD AND MATERIALS

### Study design

In the present experimental study, 24 male Wistar rats were obtained from Baqiyatallah Hospital in Tehran, Iran. The average weight of the animals ranged from 180 g to 220 g, and they were approximately 10 weeks old. The rats were randomly divided into four groups, with six rats in each group: the first group served as the control and received no treatment; the second group underwent surgical stress only, without varicocele induction (sham); the third group underwent surgery and had a varicocele model induced (VCL); and the fourth group, which had the varicocele induced, was treated by gavage with Naringenin (20 mg/kg) dissolved in a vitamin C solution (100 mg/ml) for 21 days (N20+C). All animals were maintained under standard conditions, with a 12-hour light and 12-hour dark cycle, a temperature range of 20-23°C, and free access to food and water.

### Surgery

To induce a varicocele, the animals were first anesthetized with 5% ketamine (40 mg/kg, intraperitoneally) and 2% xylazine (5 mg/kg, intraperitoneally). In the second step, the diameter of the renal vein was reduced to approximately 1 mm by ligating the left renal vein directly medial to the junction of the adrenal and spermatic veins. In the third step, the anastomotic branch between the left testicular vein and the left common iliac vein was ligated (Razi *et al*., 2011). In sham group, only laparotomy was performed after anesthesia without any ligation.

### Histological assay

After 21 days of treatment, the animals were slaughtered, and their left testes were removed. The testes were washed with normal saline, and half of them were fixed in Bouin’s fixative. Following the fixation period, the samples were dehydrated, cleared, embedded in paraffin, and sectioned using a microtome to obtain 5-6 µm thick tissue sections, which were then prepared on slides (Mobaraki *et al*., 2018).

#### Hematoxylin & Eosin staining

The glass slides were stained with H&E and mounted. The sections were examined using a light microscope to assess the quality of testicular tissue in all groups.

#### Immunofluorescence staining for Hsp70

To assess the immunoreactivity of HSP70, frozen sections were cut to a thickness of 8-10 µm and immersed in cold phosphate-buffered saline (PBS) for 5 minutes. To block non-specific binding sites of the samples, superblock solution was applied for 30 minutes. The cross sections were then incubated overnight at 4°C with a primary antibody specific to HSP70. The blocking serum alone served as the control section. After washing with PBS, the sections were incubated for 30 minutes at room temperature with a fluorescent anti-mouse secondary antibody (IgG) conjugated to Alexa Fluor® 594. Finally, the sections were counterstained with DAPI to visualize nuclear DNA in blue. The sections were examined using fluorescence microscopy (Ferro *et al*., 2017).

#### Biochemical evaluation

MDA: The MDA in homogenized tissue reacts with thiobarbituric acid (TBA) to form a red complex. Briefly, a mixture of homogenized tissue, TBA, trichloroacetic acid, and hydrochloric acid was boiled in a water bath for 40 minutes. After cooling to room temperature, the samples were centrifuged at 1000 g for 10 minutes. The absorbance of the supernatant was measured at 535 nm, and the MDA concentration (C) was calculated using the following equation (Karimi *et al*., 2020).

C = Absorbance/1.56 × 10^5^

#### SOD

SOD activity was assessed by a protocol described by Madesh and Balasubramanian (Madesh & Balasubramanian, 1998).

#### Total antioxidant capacity (TAC)

To measure the TAC, the ImAnOx colorimetric test system was utilized. The antioxidants in the samples reacted with a specific exogenous amount of ready H2O2 and reduced its concentration. The Total Mixture of Buffers (TMB) was used as a colored indicator to determine the residual H2O2. The samples were evaluated at a wavelength of 450 nm using a microtiter plate reader (Tuzgen *et al*., 2007).

### Gene expression

#### RNA extraction and cDNA synthesis

Total RNA was extracted from the testes tissue by Pars Tous Azmon kit (Iran). To confirm the extraction of RNA, we measured the concentration of extracted RNA by Nano-drop spectrophotometer. The cDNA was synthesized by Pars Tous Azmon kit (Iran).

#### Real-time PCR

After designing the primers by Gen Bank (http://www.ncbi.nlm.nih.gov) and oligo7 software, the primers were ordered. The mixture of forward and reverse primers (2 µl), SYBR Green Master Mix (10 µl) and one microliter of cDNA were reached to 20 µl by adding pure water and then real time PCR was run, and the gene expression was evaluated by Cq (Quantification cycles) values and calculated using the REST software (2009).

## RESULTS

### Hematoxylin & Eosin staining

**Figure 2:**
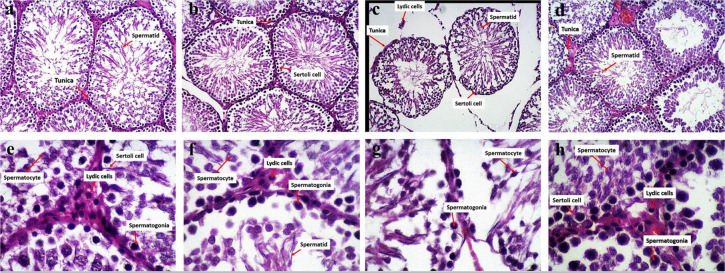
H&E staining images. a and e) Control group, b and f) Sham group: in both groups, normal shape and intact membrane of seminiferous tubules with germ cells and spermatogonial cells in different stages of development. C group: the damage of seminiferous tubules is lower than in the VCL group and higher than in the control and sham group. (a-d have 40X magnification and e-h have 100X magnification).

Based on H&E images, the seminiferous tubules in the sham and control groups exhibited a normal shape and intact membranes. Germ cells and spermatogonial cells at various stages of development displayed normal nuclear shapes and cytoplasmic characteristics, extending from the basement membrane to the center of the seminiferous tubules. Sertoli cells were situated between different stages of spermatogonial cells and spermatocytes. Leydig cells were found outside the seminiferous tubules in the interstitial space. The thickness of the germinal layer in these groups was larger than in the other groups, indicating active spermatogenesis (Figure 2a, b, e and f).

According to H&E images of the VCL group, the basal membrane of some seminiferous tubules remains intact. However, in most areas, cell death has resulted in the loss of cells and rupture of the membrane, leading to damage of the blood-testicular barrier and the infiltration of blood into the seminiferous tubules. The distance between the seminiferous tubules has increased, and the space between different germ cell lines in the VCL group is greater than in the other groups. Despite the death of many cells, various stages of spermatogonial cells were observed from the basal membrane to the center of the seminiferous tubules. Additionally, the number of Sertoli and Leydig cells has decreased in the VCL group (Figure 2c and g).

The H&E images of the N20+C group showed an increase in the free space between some seminiferous tubules and a rise in cell death compared to the sham and control groups. However, most of the tubules maintained an intact basal membrane. In certain areas, due to a decline in blood-testicular barrier function, blood droplets were observed within the seminiferous tubules. The germ cells and spermatogonial cells at various stages of development exhibited normal nuclear shapes and cytoplasmic characteristics, and their numbers, observed from the basement membrane to the center of the seminiferous tubules, were greater than those in the VCL group. Despite the presence of cell death, Sertoli cells were found in the spaces between different stages of spermatogonial cells and spermatocytes, and their absence was less pronounced than in the VCL group. Leydig cells were located outside the seminiferous tubules in the intra-tubular space, and their numbers were lower than in the sham and control groups but higher than in the VCL group (Figure 2d and h).

### Immunofluorescence staining for Hsp70

**Figure 3:**
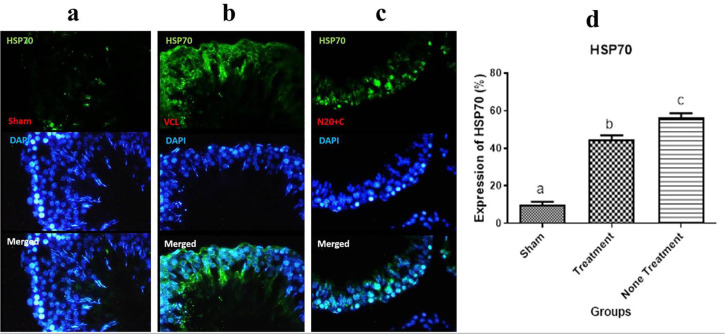
Immunofluorescence staining for Hsp70, a) Immunofluorescence image of the Sham group, the green spot that is the HSP70 expression landmark is lower than in other groups, b) Immunofluorescence image of the VCL (non-treatment) group, increase the HSP70 expression in comparison to other groups, c) Immunofluorescence image of the N20+C (treatment) group, the HSP70 expression is lower than in the VCL group and higher than in the Sham group; and d) The diagram of expression of HSP70, the expression pf HSP70 in the Sham group was lower than in the VCL (non-treatment) and the N20+C (treatment) groups (*p*<0.001), and in the (treatment) group was lower than in the VCL (non-treatment) (*p*<0.05).

The expression of HSP70 in the sham group was lower than in the VCL and N20+C groups (Figure 3a) and increase in the VCL group in comparison to the Sham and N20+C groups (Figure 3b). The expression of HSP70 in N20+C group was lower than in the VCL group and more than in the Sham group (Figure 3c). Based on the diagram of HSP70 expression, the HSP70 expression in the Sham group was lower than in the VCL and N20+C groups (*p*<0.001), and in the N20+C group it was lower than in the VCL (*p*<0.05) (Figure 3d).

### Oxidative stress

**Figure 4:**
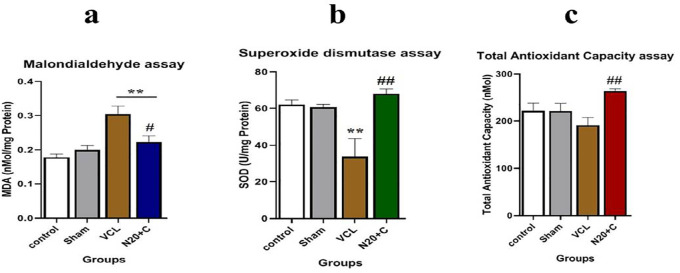
a) MDA level diagram: the MDA level in the VCL group was higher than in the N20+C group (*p*<0.01); SOD activity diagram: the SOD activity in the N20+C group was higher than in the VCL group (*p*<0.01) and c) The TAC level diagram: the TAC level in the VCL group was lower than in the N20+C group (*p*<0.01).

The MDA level in the VCL group was higher than in the N20+C group (*p*<0.01) and there was no significant difference between the Sham, Control and N20+C groups (Figure 4a). The SOD activity in the N20+C group was higher than in the VCL group (*p*<0.01) and there was no significant difference between the Sham, Control and N20+C groups (Figure 4b). The TAC level in the VCL group was lower than in the N20+C group (*p*<0.01) and there was no significant difference between the Sham, Control and N20+C groups (Figure 4c).

### Gene expression

**Figure 5:**
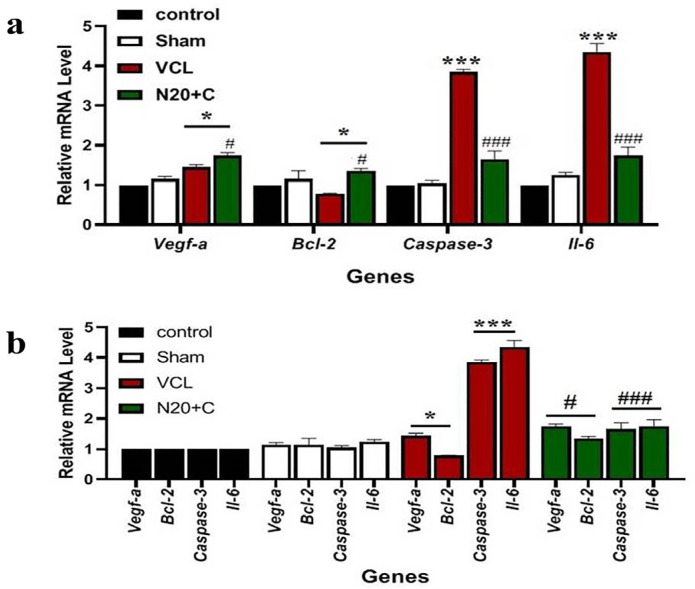
a) Comparison of genes expression between groups, the expression of VEGF-a gene in the N20+C group was higher than in the VCL group (*p*<0.05); the BCL2 gene expression in the N20+C group was higher than in the VCL group (*p*<0.05); the caspase-3 gene expression in the VCL group was higher than in the N20+C group (*p*<0.001) and the expression of the IL-6 gene in the VCL group was higher than in the N20+C group (*p*<0.001) and b). Comparing different gene expressions in each group, there was no difference in gene expression in the Control and Sham groups; in the VCL group the VEGF-a gene expression was higher than BCL-2 (*p*<0.05); and caspase-3 gene expression was lower than IL-6 (*p*<0.001); and in the N20+C group the VEGF-a gene expression was higher than BCL-2 (*p*<0.05).

The expression of VEGF-a gene in the N20+C group was higher than in the VCL group (*p*<0.05) and there was no significant difference between the Sham and VCL groups (Figure 5a). The expression of BCL2 gene in the N20+C group is more than in the VCL group (*p*<0.05) and there was no significant difference between the Sham group and the N20+C and VCL groups (Figure 5a). The expression of caspase-3 gene in the VCL group was higher than in the N20+C group (*p*<0.001) and there was no significant difference between the Sham group and the N20+C group (Figure 5a). The expression of IL-6 gene in the VCL group is more than in the N20+C group (*p*<0.001) and there was no significant difference between the Sham group and the N20+C group (Figure 5a). There was no significant difference in VEGF-a, BCL-2, caspase-3 and IL-6 genes expression between the Control and the Sham groups (Figure 5b). In the VCL group, the VEGF-a gene expression was higher than in the BCL-2 (*p*<0.05) and caspase-3 gene expression was lower than the IL-6 (*p*<0.001) (Figure 5b). In the N20+C group the VEGF-a gene expression was higher than BCL-2 (*p*<0.05) (Figure 5b).

## DISCUSSION

The prevalence of infertility is increasing worldwide, with approximately 15% of couples affected. One of the most significant causes of male infertility is varicocele, which affects nearly 35% of men with primary infertility and 81% of men with secondary infertility (Gorelick & Goldstein, 1993). Varicocele decreases the quality of semen parameters, but the exact mechanism of this fact is unknown (Eisenberg & Lipshultz, 2011). It may occur because a varicocele increases blood stagnation in the testicular veins, leading to elevated temperatures in the testicles and the occurrence of oxidative stress, which impairs spermatogenesis (Mallidis *et al*., 2011).

In the present study, we demonstrated that varicocele adversely affects spermatogenesis and reduces the number of spermatogonial cells at all stages, thereby confirming the validity of the varicocele animal model. Other studies have shown that varicocele damages the testes and disrupts spermatogenesis in both humans (Kang *et al*., 2022) and animal (Razi & Malekinejad, 2015) models. The administration of naringenin in rats may reduce the damage caused by varicocele, improving the quality of testicular tissue, and preserving spermatogonial cells at all stages. The study on the protective effects of naringenin confirms our findings, demonstrating that naringenin administration enhances testicular tissue quality, sperm parameters, and serum testosterone levels, while reducing apoptosis in Sertoli and Lydig cells in diabetic mice (Roy *et al*., 2013).

The HSPs are an endogenous family of protective proteins found in both the nucleus and cytoplasm, and they are essential for normal cellular function. ROS, cytotoxic lysosomal enzymes, and cytoskeletal changes can activate the expression of HSPs (Afiyani *et al*., 2014). HSP70 act as apoptosis inhibitor in cells and can regulate apoptotic cellular signaling (Liao *et al*., 2006). HSP70 as chaperones assist the unfolding and assembly of proteins in the cytoplasm, the mitochondria and the endoplasmic reticulum (Kaur & Bansal, 2003). According to the immunofluorescence staining image, varicocele increased the expression of HSP70 in the testis. Furthermore, varicocele decreased SOD and enlarged the testis, thereby elevating oxidative stress. In a varicocele-induced animal model, the HSP70 gene expression was found to be increased (Hassanpour *et al*., 2017); TAC, SOD and GSH decreased and MDA increased, so oxidative stress occurred in the testes (Khosravanian *et al*., 2014). The expression of HSP70 in the N20+C group was significantly lower than in the VCL group but higher than in the Sham group. Additionally, the MDA level in the N20+C group was lower than in the VCL group, while the levels of SOD and TAC were higher than those in the VCL group. Naringenin administration mitigated the oxidative stress induced by varicocele, leading to a decrease in the expression of HSP70 - a stress response protein. A study evaluating the protective effect of Naringenin against cadmium toxicity in the testes demonstrated that naringenin administration reduced oxidative stress by increasing the levels of TAC, SOD, GSH, and catalase; while decreasing MDA levels, thereby completely preventing testicular damage (Wang *et al*., 2021).

The elevated levels of IL-6, a pro-inflammatory cytokine, along with ROS increase in infertile men with varicocele. This rise may provide insight into the pathophysiology of infertility in these patients (Nallella *et al*., 2004). In the present study, the IL-6 gene expression was increased in the VCL group compared to healthy rats; and such increase was in line with oxidative stress. VEGF is an angiogenic peptide that mediates angiogenesis and vasculogenesis. Based on some studies, VEGF is an important factor to decrease apoptosis after varicocele in rats, and it can cure the testicular damage (Tek *et al*., 2009). The expression of the VEGF gene in rats with varicocele is increased, which may help reduce apoptosis and testicular damage in the VCL group. According to the results, the expression levels of the VEGF and IL-6 genes were higher in the naringenin-treated group, compared to the control and sham groups, although they were lower than those in the VCL group. Other studies have confirmed our findings, indicating that naringenin reduces pro-inflammatory cytokines such as IL-6; thereby decreasing oxidative stress in damaged tissues (Al-Rejaie *et al*., 2015, Dou *et al*., 2013) and reduces apoptosis by increasing VEGF levels (Oguido *et al*., 2017).

There are two important pathways for apoptosis in cells: the extrinsic pathway, known as the death receptor pathway, in which Fas and caspase-8 play significant roles, and the intrinsic pathway, referred to as the mitochondrial pathway, where Bcl-2 and caspase-9 are critical in regulating apoptosis (Hongmei, 2012). In the present study, the BCL-2 gene expression was decreased, while the expression of the caspase-3 gene increased following varicocele induction in animals. This indicates that varicocele enhances apoptosis in the testes. The increase in apoptosis in the testis is primarily mediated by the intrinsic pathway, with BCL-2 and caspase-3 playing significant roles. Additionally, the TUNEL assay confirmed that apoptosis increased after varicocele induction (Lee *et al*., 2009). The administration of naringenin in rats with varicocele decreased the expression of the caspase-3 gene and increased the expression of the BCL-2 gene compared to the varicocele group. In the N20+C group, the expression levels of BCL-2 and caspase-3 were not significantly different from those in the control and sham groups, indicating that naringenin may protect the testes from apoptosis induced by varicocele. Naringenin reduces tissue damage caused by apoptosis resulting from oxidative stress, as it can modulate mitochondrial dysfunction, repair the mitochondrial membrane potential, and prevent the translocation of apoptotic proteins to the nucleus, which can induce DNA damage. Consequently, it can halt the apoptotic signaling cascade (Kapoor & Kakkar, 2014). In the VCL group, the IL-6 gene expression was higher than that of the caspase-3 gene, while the expression of the BCL-2 gene decreased in comparison to both IL-6 and caspase-3 gene expressions. This indicates that oxidative stress occurs first, followed by the induction of apoptosis, resembling the intrinsic pathway of apoptosis. Treatment with naringenin resulted in decreased expression of the IL-6 and caspase-3 genes, while increasing the expression of the BCL-2 gene. Therefore, naringenin may prevent apoptosis through the intrinsic pathway. Given that varicocele induces apoptosis via this pathway, Naringenin is a promising option for reducing apoptosis signaling in testicular tissue.

## CONCLUSION

The findings of this study suggest that naringenin may serve as a promising therapeutic agent for mitigating varicocele-induced testicular damage. Naringenin, a flavonoid with established anti-apoptotic and antioxidant properties, has the potential to restore testicular function and enhance fertility outcomes in individuals afflicted with varicocele. The ability of Naringenin to reduce oxidative stress and prevent apoptosis in testicular cells highlights its potential role in clinical settings, particularly for patients who are seeking non-invasive treatment options. Future clinical trials are warranted to evaluate the efficacy and safety of naringenin supplementation in human subjects with varicocele, as well as to establish optimal dosing regimens.
